# Evaluation of Quantitative PCR (qPCR) *Paenibacillus larvae* Targeted Assays and Definition of Optimal Conditions for Its Detection/Quantification in Honey and Hive Debris

**DOI:** 10.3390/insects9040165

**Published:** 2018-11-16

**Authors:** Franca Rossi, Carmela Amadoro, Addolorato Ruberto, Luciano Ricchiuti

**Affiliations:** 1 Istituto Zooprofilattico Sperimentale dell’Abruzzo e del Molise “G. Caporale”, Via Campo Boario 1, 64100 Teramo, Italy; a.ruberto@izs.it (A.R.); l.ricchiuti@izs.it (L.R.); 2 Medicine and Health Science Department “V. Tiberio”, University of Molise, Via de Santis, 86100 Campobasso, Italy; carmela.amadoro@unimol.it

**Keywords:** *Paenibacillus larvae*, optimized qPCR, quantification, honey, hive debris

## Abstract

The application of quantitative PCR (qPCR) as a routine method to detect and enumerate *Paenibacillus larvae* in honey and hive debris could greatly speed up the estimation of prevalence and outbreak risk of the American foulbrood (AFB) disease of *Apis mellifera*. However, none of the qPCR tests described so far has been officially proposed as a standard procedure for *P. larvae* detection and enumeration for surveillance purposes. Therefore, in this study, inclusivity, exclusivity and sensitivity of detection of *P. larvae* spores directly in samples of honey and hive debris were re-evaluated for the previously published qPCR methods. To this aim, recently acquired *P. larvae* sequence data were considered to assess inclusivity in silico and more appropriate non-target species were used to verify exclusivity experimentally. This led to the modification of a previously described method by shortening the forward primer, designing a new reverse primer and using more stringent amplification conditions. The new test allowed the detection of *P. larvae* spores in honey and hive debris down to 1 CFU/g. The qPCR test optimized in this study proved suitable for quantification and also for identification of field *P. larvae* strains and real contaminated samples. Therefore, it is proposed for reliable detection and quantification of *P. larvae* in honey and hive debris, thus circumventing the disadvantages of late AFB diagnosis based on clinical symptoms and possible underestimation of spore numbers that is the main drawback of culture-dependent procedures.

## 1. Introduction

*Paenibacillus larvae* is the causative agent of American foulbrood (AFB), the most destructive and highly contagious disease of the honey bee (*Apis mellifera*) that infects larvae during the first 48 h after egg etching [1]. Notification of AFB to the veterinary authority is mandatory in many countries and its diagnosis and official outbreak registration is based on the observation of clinical symptoms [2,3].

*P. larvae* endospores are the infective form of the bacterium that resist high temperatures and antimicrobial agents and can persist in hives for decades [4]. Their spread occurs via bee products, e.g., honey, equipment from infected hives and the robbing behavior of bees [5,6].

Diagnosis based on clinical symptoms does not efficiently prevent AFB spread since the bacterium might have already been transmitted through the above-mentioned routes.

Therefore, the application of diagnostic procedures allowing to detect early and quantify the bacterium in substrates like honey and hive debris could help to identify apiaries with a high risk of infection, thus allowing the prevention of clinical manifestation of the disease and further spread of *P. larvae* spores. Monitoring hive debris is current practice in some countries, such as Czech Republic, though by cultural methods [7]. Therefore, providing analytical tools that facilitate *P. larvae* detection can make the prevention of AFB spread more efficient.

The usefulness of enumerating *P. larvae* in honey is justified by the existence of a positive correlation between the presence and number of spores and the prevalence of AFB outbreaks in apiaries. Pernal and Melathopoulos [8] associated a prevalence of 1–5% in apiaries to beekeepers whose honey samples contained approximately 1000 CFU/g of spores, while 500 CFU/g of spores or lower were not always associated to AFB outbreaks. 

One study regarding the correlation between the number of *P. larvae* spores in hive debris and AFB clinical manifestations was carried out by Carpana [9], who found that the number of *P. larvae* spores in hive debris and the percentage of AFB cases were strongly correlated. Clinical symptoms ranged between 8% of hives for apiaries with less than 1000 CFU/g spores and 78% of hives for apiaries with 100,000 CFU/g of spores in hive debris. Forsgren and Laugen [10] observed that samples of debris can reveal the AFB infection in course in the bee colony. Moreover, in the debris *P. larvae* spores accumulated during time, thus allowing the a posteriori diagnosis of acute infection episodes and the identification of hives more at risk of spreading the infection.

Therefore, not just presence but also the number of *P. larvae* spores in honey and hive debris is an indicator of AFB prevalence and outbreak risk. Consequently, its determination by rapid methods would be of great support in AFB containment.

Cultural methods used to enumerate *P. larvae* spores are time consuming, not completely selective and need confirmation by isolate identification. Moreover, differences among biotypes in resistance to the heat treatments used to kill vegetative cells prior to enumeration and in the germination rate determines an underestimation of spore numbers [11]. Therefore, qPCR can be the only reliable method to quantify *P. larvae* in hive associated samples.

Despite different qPCR methods developed for this purpose, none of them has been recommended for the direct detection and enumeration of *P. larvae* in hive associated materials [12,13]. Four qPCR tests targeted on the *P. larvae* 16S rRNA gene were described for rapid identification and early detection of this bacterium. Han et al. [14] developed an ultra-rapid amplification method and applied it to enumerate *P. larvae* vegetative cells in AFB infected larvae for early diagnosis. Chagas et al. [15] proposed a method for the unequivocal identification of presumptive *P. larvae* isolates. The qPCR test designed by Martínez et al. [16] allowed for detecting as little as 2 *P. larvae* spores/g in honey and 10^3^ CFU/g in hive debris [10]. Quintana et al. [17] designed a qPCR test able to detect as little as 28 *P. larvae* spores in larval scales. In addition, a *P. larvae*-specific qPCR assay was included in a triplex test aimed at the qualitative detection of the microorganism in brood samples [18]. Quantification of *P. larvae* by qPCR was not applied to honey and hive debris so far. 

To this end, this study was carried out to select the most suitable *P. larvae*-specific qPCR method among those already described. Since only a few gene sequences were available for *P. larvae* and strictly related microorganisms when most primers used in those assays were designed, their inclusivity and exclusivity was re-assessed. These aspects were evaluated in silico and experimentally in this study. Based on the results obtained, it was deemed opportune to modify or design new primers and optimize amplification conditions to make qPCR detection/quantification of *P. larvae* in honey and hive debris more sensitive and accurate.

## 2. Materials and Methods

### 2.1. Bacterial Strains and Culture Conditions

Reference bacterial strains used in this study were *P. larvae* ATCC 9545, *P. naphthalenovorans* DSM 14203, *P. glucanolyticus* DSM 5162 and *P. chitinolyticus* DSM 11030. In addition, 50 *P. larvae* isolates previously identified with the end point PCR test with primers AFB-F/AFB-R [19], were used to experimentally confirm the inclusivity of the new test. All the strains were grown on *Paenibacillus larvae* agar (PLA), in which all the *Paenibacillus* species tested grew well, prepared as described by Schuch et al. [20] with components from Sigma Aldrich (Milan, Italy), or on Sheep Blood Agar (Biolife Italiana, Milan, Italy) incubated at 37 °C for 2–5 days in the presence of 9% CO_2_. All the reference strains were checked for purity by streaking on Sheep Blood Agar plates before extracting DNA.

To prepare qPCR standards from known numbers of *P. larvae* spores, colonies were harvested by adding 2 mL of phosphate buffered saline (PBS, 8.0 g/L NaCl, 0.2 g/L KH_2_PO_4_, 2.9 g/L Na_2_HPO_4_, 0.2 g/L KCl, pH 7.4) and scraping with an “L” shaped sterile plate spreader from Sheep Blood Agar plates kept at room temperature for 30 days after bacterial growth. At this time no vegetative cells were visible in the suspensions by microscope observation of slides stained with a 3 g/L crystal violet (BioMerieux Italia, Bagno a Ripoli, FI, Italy) water solution. The spore suspensions were heat-shocked at 80 °C for 10 min to kill the remaining vegetative cells and soon cooled down by incubation at −20 °C for 5 min. The heat-treated spore suspensions were centrifuged at 10,000 rpm for 5 min, washed twice with 2 mL of sterile PBS and serially diluted to determine their number on PLA medium and artificially inoculate honey and hive debris. Spore suspension dilutions were stored at −20 °C for up to six months prior to sample inoculation if not used immediately.

The types of inoculated samples were 0.5 g/mL honey suspensions and 100 µg/mL hive debris suspensions in deionized water sterilized by autoclaving at 121 °C for 15 min.

### 2.2. DNA Extraction

Crude DNA extracts were prepared from *Paenibacillus* spp. bacteria by re-suspending a single colony picked with a sterile loop in 100 µL of sterile 10 mM Tris/HCl buffer, pH 8.0, and heating at 100 °C for 5 min. The suspension was centrifuged at 8000 rpm for 5 min and the clear supernatant was used in qPCR reactions.

DNA extraction from honey and hive debris artificially inoculated with decimal dilutions of spore suspension to obtain final spore numbers in the range 0.1–10^6^ CFU/g for honey and in the range 1–10^7^ CFU/g for hive debris, was carried out from 2 mL of sterilized honey suspension or 1 mL of hive debris suspension. Real samples were processed by preparing 1:2 (*w*/*v*) honey suspensions and 1:10 (*w*/*v*) suspensions of hive debris, adult bee or brood cells with larval scales homogenized with a sterile pestle in sterile deionized water prior to DNA extraction. The DNA extraction was carried out with the NucleoSpin Tissue kit (Macherey-Nagel GmbH & Co. KG, Düren, Germany) as follows: the inoculated honey and hive debris suspensions were centrifuged at 14,000 rpm for 2 min and the pellets were resuspended in 90 µL of T1 buffer µL added with 10 of µL proteinase K. The samples were incubated for 1 h at 56 °C. To the suspensions T1 buffer was added to reach the volume of 205 µL and these were centrifuged at 12,000 rpm for 10 min. The supernatant was transferred in a new sterile tube and the extraction was prosecuted according to the NucleoSpin Tissue kit instructions that follow proteinase K treatment. DNA was finally re-suspended in 20 µL of elution buffer by a two-step elution with 10 µL of the same buffer.

### 2.3. In Silico Analysis of Primer Specificity and Inclusivity and Primer Design

The exclusivity of the oligonucleotides previously proposed for *P. larvae* detection by qPCR [13,14,15,16,17] was verified as follows: (i) the bacterial species with highest identity of the 16S rRNA gene sequence with *P. larvae* were identified by BLAST analysis (https://blast.ncbi.nlm.nih.gov) run in “megablast” mode and excluding the “*Paenibacillus larvae*” taxon, (ii) the 16S rRNA genes of the identified species and of *Paenibacillus* species known to be associated to hive matrices were aligned by Clustal Omega (http://www.ebi.ac.uk/Tools/msa/clustalo/), (iii) the positions of the previously designed primers were determined in the aligned sequences to analyze matching with the corresponding region in *P. larvae*.

To determine primer inclusivity, the 16S rRNA gene sequences of 90 *P. larvae* isolates available in the nucleotide database (https://www.ncbi.nlm.nih.gov/nucleotide) and in the Ribosomal Database Project (RDP; https://rdp.cme.msu.edu/), plus all the 16S rRNA genes found (eight in each) in the eight *P. larvae* completely assembled genomes, comprising all four ERIC types, and six 16S rRNA genes of a not completely assembled genome of strain *P. larvae* DSM 25719 (Acc. N. NZ_ADFW00000000), were aligned by Clustal Omega (https://www.ebi.ac.uk/Tools/msa/clustalo/). The target gene region of primers PL-F and PL-R designed by Dainat et al. [18] was defined by BLAST analysis.

### 2.4. PCR Amplification

PCR was carried out in 20 µL reactions with the KapaSybr Fast qPCR Master Mix (KapaBiosystems, Sigma-Aldrich, Milan, Italy). Two µL of DNA and of each primer were added and nuclease-free water to reach the reaction volume. The qPCR programs were run in a QuantStudio 5 thermal cycler (Applied Biosystems, Thermofisher Scientific, Rodano, MI, Italy).

PCR with primer pair Pltr-F/Pltr-R was carried out as previously described [15]. Moreover, the method was modified to be more specific by using primers in 0.25 µM concentration, decreasing the number of cycles from 40 to 36 and increasing the annealing temperature from 60 °C to 64 °C, while the annealing time was decreased from 1 min to 13 s.

Primers PL2-Fw/PL2-Rev, were used in the conditions described by Martínez et al. [16].

Forward primers PLAup and PLAup2, and reverse primer PLAdw were designed in this study and are reported in Table 1.

In the PCR test, optimized to be fully exclusive while at the same time maintaining high sensitivity, primers PLAup and PLAup2 were used in 0.25 µM concentration, while PLAdw was used in 0.15 µM concentration. The PCR program comprised initial denaturation at 94 °C for 4 min, 40 cycles of denaturation at 95 °C for 15 s and annealing at 56 °C for 10 s followed by melting curve analysis.

PCR reactions with the universal bacterial primer pair HDA1/HDA2 [21], aimed at revealing the presence of bacteria, thus allowing evaluation of the quality of DNA extracts, were carried out for each DNA sample. Both primers were used in 0.25 µM concentration in PCR programs comprising initial denaturation at 94 °C for 4 min, 40 cycles of denaturation at 95 °C for 15 s and annealing at 63 °C for 10 s followed by melting curve analysis. Samples presenting amplification and a sharp melting peak at a temperature ranging between 82 and 87 (dissociation temperature of the amplicon is expected to vary for different bacterial species that can be present) were retained for the *P. larvae* targeted assay.

No template control (NTC) reactions were run in each qPCR experiment and Ct >40 was taken as threshold for “no amplification”.

### 2.5. 16S rRNA Sequencing

All the DNA extracts from single colonies of the reference strains were submitted to species confirmation by sequencing of the 16S rRNA gene.

The 16S rRNA gene amplification was carried out as described by Weisburg et al. [22] with primers fD2/rD1 re-designed without 5′ linker sequence.

Amplification products were purified by the Wizard SV Gel and PCR Clean-Up System (Promega, Madison, WI, USA) and sequenced on both directions with the same primers by GATC Biotech (Constance, Germany).

## 3. Results

### 3.1. In Silico Analysis of Primer Exclusivity

The oligonucleotide pairs previously proposed for the detection of *P. larvae* by qPCR were re-assessed in silico for exclusivity. The primer pair designed by Dainat et al. [18] was not included in the analysis since BLAST alignment showed that it is targeted on phage DNA present in all *P. larvae* genomes but in a highly variable number of copies that ranges between tens and hundreds. Therefore, the cell number cannot be determined because there is not a stable correspondence between the target region copy number and the number of cells, though the method is supposed to be the most sensitive among those available for presence/absence determinations.

The first step was identifying the bacterial species most closely related to *P. larvae* at the 16S rRNA gene sequence level. These were identified by BLAST analysis using as query the 16S rRNA gene locus BXP28_01730 of *P. larvae* ATCC 9545, GenBank Acc. N. CP019687. The species most closely related to *P. larvae* were *P. naphthalenovorans* and *P. chitinolyticus* with 95% identity of the 16S rRNA sequence with *P. larvae*. These species and others sharing 94% identity of the 16S rRNA sequence with *P. larvae*, as well as *Paenibacillus* spp. ubiquitous or found to occur in hive matrices, namely *P. glucanolyticus*, *P. alvei* and *P. apiarius* [23], were aligned by Clustal Omega to analyze the sequence identities at the annealing sites of the qPCR *P. larvae* targeted primers previously described.

It appeared that, with no exception, the previously reported PCR tests used reverse primers annealing at sites either identical or differing at most of two nucleotides in internal sites between *P. larvae* and the other species considered, while the forward primers were specific for *P. larvae*. Moreover, among the reverse primers, 16SNR [14], was found to lack a “C” nucleotide corresponding to position 323502 of the *P. larvae* ATCC 9545 genome GenBank acc. CP019687, 16S rRNA locus tag BXP28_01730 and present in all the *P. larvae* 16S rRNA gene sequences analyzed.

**Figure 1:**
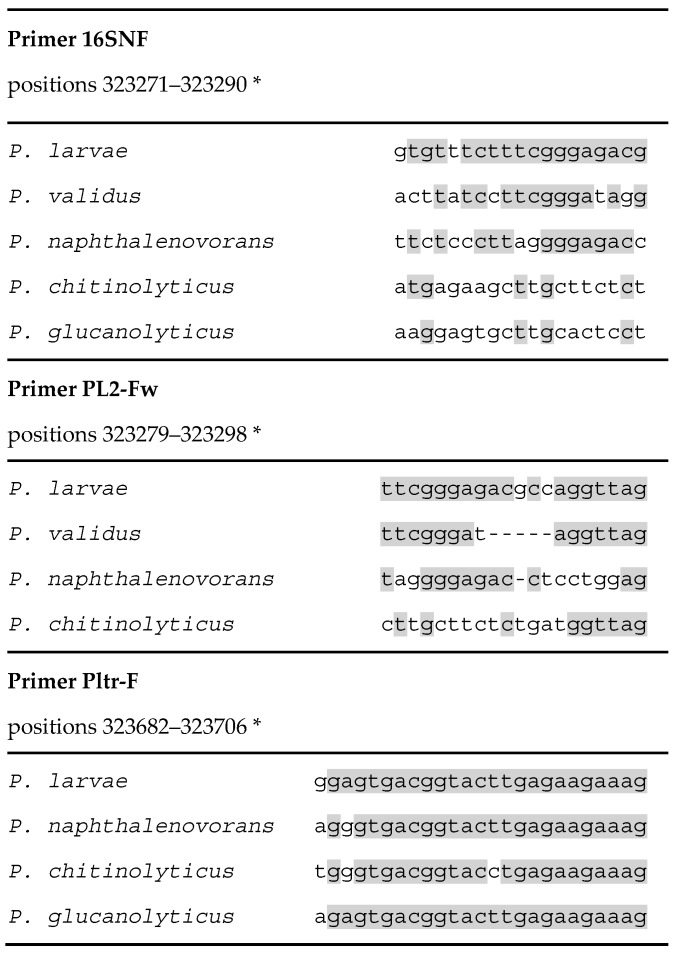
Sequence alignments of the annealing sites of forward primers from *P. larvae* targeted identification and detection qPCR assays in *P. larvae* and closely related or hive associated *Paenibacillus* species. All the types of matching observed are shown and positions matching between *P. larvae* and at least one of the other species are shadowed. The aligned sequences have accession numbers NR_112053, AB073189, NR_028817 and AB073203 for *P. chitinolyticus*, *P. glucanolyticus, P. naphthalenovorans* and *P. validus*, respectively. * Positions in the *P. larvae* ATCC 9545 genome GenBank Acc. n. CP019687, locus tag BXP28_01730.

The forward primers showed different degrees of identity with the corresponding regions in other species. Figure 1 shows all the different types of sequence matching of the forward primers observed with non-target species.

The forward primer PL 167 fw [17] was not reported in Figure 1 since it is identical to primer PL2-fw but with three more nucleotides at the 5′ terminus, and one nucleotide less at the 3′ terminus. The three first nucleotides at the 5′ terminus of this primer are identical in all the species compared, except for some *P. larvae* strains in which the first nucleotide is “T”. 

### 3.2. In Silico Analysis of Primer Inclusivity

A BLAST alignment of all the 16S rRNA gene sequences available for *P. larvae* was carried out to analyze the intra-species variability at the annealing sites of the primers considered, in order to define their inclusivity for all *P. larvae* strains.

To this aim, all the eight 16S rRNA genes found in each *P. larvae* genome and other 90 *P. larvae* 16S rRNA gene sequences available in the public domain database were aligned by Clustal Omega.

For one of those primers, i.e., 16SNF [14], an intra-genome and intra-species 16S rRNA gene sequence variability was observed. One mismatch at position 8 of the primer, consisting in a “C” to “T” transition was observed in most cases. Moreover, the insertion of a “T” nucleotide was observed at the same position for two strains. Strain *P. larvae* Ymb1 (Acc. N. EF187246) has two mismatches with the primer 16SNF, while *P. larvae* PL75 (Acc. n. KU682820) has a deletion corresponding to position 6 of the primer. Concerning intra-genome variability, for *P. larvae* Eric_I (Acc. n. CP019651) three 16S rRNA genes vary in one position and one in two positions of the 16SNF primer annealing site, for *P. larvae* ATCC 9545, ATCC 13537 (Acc. n. CP019794), CCM 38 (Acc. n. CP020327), Eric_III (Acc. n. CP019655) and Eric_IV (Acc. n. CP019659) five 16S rRNA genes vary in one position, for *P. larvae* SAG 10367 (Acc. n. CP020557) all 16S rRNA genes vary in one position, while strain DSM 25430 (Acc. n. NC_023134) has one mismatch with the primer in only one 16S rRNA gene.

The above described mismatches are frequent in *P. larvae* strains since they were found in about 35% of the 16S rRNA genes analyzed. Moreover, intra-genome variability in this region was also high. Notably, the annealing site of primer 16SNF is contained in or overlapping to the annealing sites of forward primers used in conventional PCR test designed by Govan et al. [24] and Dobbelaere et al. [25] that are currently considered the gold standard for *P. larvae* detection and identification [11] and in the conventional PCR test designed by Piccini et al. [26]. The presence of mismatches in the annealing sites of these primers could reduce the PCR efficiency, an effect that increases with the number of mismatches [27].

The other forward primers analyzed (Figure 1) did not present mismatches with any *P. larvae* 16S rRNA gene and therefore were experimentally evaluated for specificity against *Paenibacillus* species not previously tested and closely related to *P. larvae*, namely *P. naphthalenovorans* and *P. chitinolyticus*, and against *P. glucanolyticus* as a representative of the ubiquitous *Paenibacillus* species with best matches of the primer annealing sites with *P. larvae*.

### 3.3. Experimental Evaluation of Exclusivity and Sensitivity the qPCR Tests

Exclusivity was re-evaluated by using crude DNA extracts obtained from single colonies of all the bacterial strains used in this study.

The qPCR tests proposed by Chagas et al. [15] gave amplification products at low Ct values, e.g., 18–22, from the non-target species even when PCR conditions were made as stringent as possible by using primers at 0.25 µmol/L concentration, much lower than indicated by the authors, and by increasing the annealing temperature from 60 °C to 64 °C. Moreover, all the non-target species presented a melting peak at the same temperature of that given by *P. larvae* ATCC 9545, and therefore could generate false positives in isolate identification and in the direct detection of *P. larvae* from hive associated matrices.

Primers PL2-Fw/PL2-Rev, when used in the conditions described by Martínez et al. [16], gave primer dimers in the no template control and in reactions with non-target species, according to what was also reported by the authors. Moreover, amplification with Ct 38 and a melting peak that could be confused with the amplification product from *P. larvae*, appeared for *P. naphthalenovorans* and *P. chitinolyticus*. This could generate uncertain results when colonies of bacterial isolates are analyzed for identification.

Specificity was improved by using PL2-Fw paired with a new reverse primer, PLAdw (Table 1), designed in this study to be specific for *P. larvae* in order to improve exclusivity, and increasing the annealing temperature to 60 °C. To avoid primer-dimer formation, the primer PLAdw was used at 0.25 µM concentration. In these conditions 10^2^ and 10 CFU/g of *P. larvae* spores could be detected in artificially inoculated hive debris and honey, respectively.

The reverse primer PLAdw can present mismatches consisting in a “G” to “A” transition in two positions that correspond to nucleotides 323391 and 323407 in the genome of *P. larvae* ATCC 9545 and are located at 6 and 21 nucleotides from its 3′ terminus, respectively. These transitions never occur together in the same gene. Only one strain, namely *P. larvae* ATCC 9545, was found to have the mutation at the position corresponding to nucleotide 6 of primer PLAdw, but in only one of its eight 16S rRNA gene copies. The mutation at the position corresponding to nucleotide 21 of primer PLAdw was found for four strains in two and for one strain in three 16S rRNA gene copies, respectively. These mutations were not observed in all other available *P. larvae* 16S rRNA sequences. Therefore, the primer PLAdw was designed without degenerated positions, considering that the above described mutations are not frequent, being observed respectively in 0.01% and 0.15% of the 16S rRNA gene sequences in *P. larvae* genomes.

### 3.4. Design of Modified P. larvae Specific Forward Primers

To ensure exclusivity, primer PL2-Fw was shortened of one nucleotide at its 3′ terminus and the resulting primer was labeled as PLAup (Table 1).

Moreover, considering the good specificity for *P. larvae* of the 16SNF primer annealing site (Figure 1), a second forward primer, PLAup2, with annealing site overlapping to that of 16SNF, but with degenerated positions corresponding to the variable nucleotide positions observed, was designed in this study (Table 1).

Forward primers PLAup and PLAup2, and the reverse primer PLAdw are theoretically able to anneal to *P. larvae* strains of all four ERIC types.

### 3.5. Optimization of New qPCR Tests for P. larvae

PCR cycle and primer concentration were optimized by testing different amplification conditions for the two primer pairs PLAup/PLAdw and PLAup2/PLAdw. Maximum sensitivity was reached for both primer pairs when an annealing temperature of 56 °C and a concentration of 0.25 µM of the forward primer and 0.15 µM of reverse primer were used. The Ct values obtained for the same samples inoculated with known *P. larvae* ATCC 9545 spore numbers was found to be comparable for the two primer pairs and the lowest number of *P. larvae* spores detected was 1 CFU/g in honey and hive debris for both. However, the latter primer pair gave amplification at Ct 37 from the non-target species *P. naphthalenovorans* and *P. glucanolyticus* of PCR products with melting peaks that could be confused with the *P. larvae* specific peak. For this reason, the primer pair PLAup/PLAdw was selected for the detection of *P. larvae* directly in samples and for the construction of calibration curves for its quantification in honey and hive debris.

### 3.6. Quantification of P. larvae in Honey and Hive Debris

**Figure 2:**
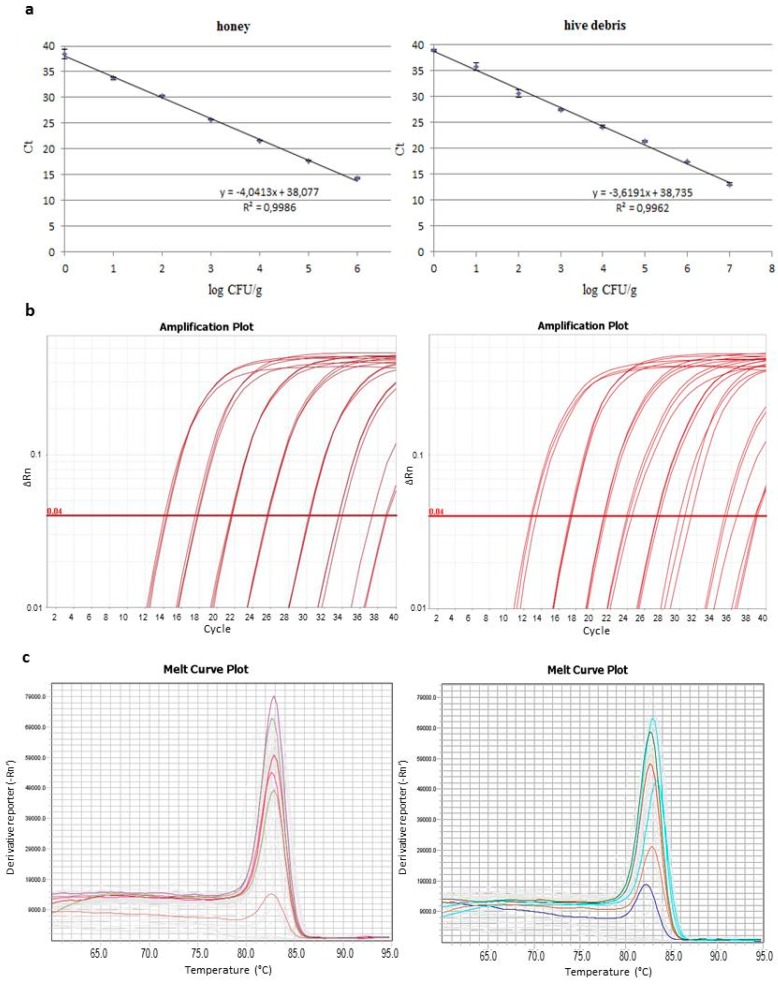
(**a**) Calibration curves used for the quantification of *P. larvae* spores in honey and hive debris by the qPCR test with primers PLAup/PLAdw: Ct values, defined on the automatic threshold, are the average of those from three replicate reactions; (**b**) corresponding amplification curves; (**c**) melting curves of the amplification products obtained from one series of standards for each sample type.

Calibration curves were constructed by plotting Ct values against CFU/g in samples of honey and hive debris artificially inoculated with spores of *P. larvae* ATCC 9545 in known numbers. Examples of those curves constructed by using three replicates of DNA extracts for each point are given in Figure 2, with the corresponding amplification and melting curves. The linearity range encompassed the whole set of spore numbers tested and the “R” coefficient was high for both honey and hive debris. The limit of detection (LOD) and the Ct variation at lower template concentrations for the qPCR assay and for both analyzed matrices were determined to comply with the minimum information for publication of qPCR experiments (MIQE, [28]). LOD was defined from 20 amplification reactions per sample type on DNA from samples inoculated with 10 and 1 CFU/g of spores and was expressed as number of spores/g rather than target DNA copy number. A LOD of 10 CFU/g of spores was determined for both honey and hive debris. For the same reactions Ct variation at the lowest template concentration tested, i.e., 1 spore CFU/g was between 38 and 40 for both honey and hive debris. At this low concentration the probability to detect positive samples was 87% and 62% for honey and hive debris, respectively.

The method of DNA extraction and the qPCR assay with primers PLAup/PLAdw were applied to analyze hive debris, honey from a honey comb, adult bees and brood cells with larval scales from two AFB outbreaks occurring in two experimental apiaries. Moreover, this method was used to analyze DNA samples extracted from 50 field strains identified in a previous study [19]. With no exception, positive results were obtained from all these sources. The number of CFU/g of spores estimated from calibration curves was higher than 10^7^ for brood cells with larval scales, in average 10^3^ for adult bees and 10^6^ for hive debris. Therefore, demonstration was achieved that the PCR test with PLAup/PLAdw optimized in this study would allow a rapid, quantitative screening of apiaries in AFB monitoring plans.

## 4. Discussion

The choice of qPCR tests that are fully inclusive for the target species and exclusive for closely related microorganisms is crucial for obtaining reliable results from analytical procedures applied in pathogen detection and quantification directly from samples.

Based on the results of this study, the verification of previously proposed methods by a preliminary analysis of primer specificity using BLAST is necessary and can allow selection of the best performing tests among those available that can be further optimized. In particular, it was evidenced that some of the primers used in the available *P. larvae* targeted tests did not sufficiently discriminate other *Paenibacillus* species or have mismatches with the respective annealing site in all or in some *P. larvae* strains, thus making some of the available protocols unsuitable for adoption as a qPCR test for *P. larvae* detection/quantification.

The sensitivity of the qPCR method optimized in this study, that is an evolution of the method of Martínez et al. 2010 [16] in which the forward primer was shortened, the reverse primer was substituted by a *P. larvae* selective one and the amplification conditions made more stringent, is comparable or higher than for methods previously applied to honey or hive debris. Indeed, Martínez et al. (2010) [16], who did not specify if their LOD was obtained according to the MIQE guidelines [28], analyzed only honey and could detect 2 CFU/g of spores, while in this study 1 CFU/g of each matrix could be detected, though with a percentage of positive results lower than the 95% value required for LOD definition. Forsgren et al. (2014), who applied the qPCR method of Martínez et al. (2010) [16] could detect 10^3^ CFU/g of spores in hive debris. Detection of *P. larvae* spores in hive debris was carried out also by Ryba et al. (2009) [29] with an end-point PCR method that could detect 10^5^ CFU/g of *P. larvae* spores. The higher sensitivity of the assay described here is most probably attributable to the efficiency of DNA extraction.

When adopting a PCR test for diagnostic purposes it is opportune to verify if the non-target organisms used to assess the specificity of PCR methods were chosen according to correct criteria that are taxonomical relatedness, degree of sequence matching at the primer annealing site and occurrence in the same ecological niche.

A BLAST analysis of the 16S rRNA gene of *P. larvae* evidenced that strictly related microorganisms possibly present in hive associated matrices, that could generate false positives in direct analysis of samples, were not tested as non-target species when the qPCR methods were designed. Indeed, for most of the *P. larvae*-specific qPCR tests previously designed *P. alvei* was the microorganism most closely related used to assess exclusivity [14,15,16]. However, *P. naphthalenovorans* and *P. chitinolyticus* have a better matching with the *P. larvae* targeted primers compared to *P. alvei.* These species can be both present in honey and pollen, as stated in the description of the isolation sources for sequences with accession numbers KJ638115 and MG650019, so that it was deemed more correct to use them to assess the specificity of *P. larvae* targeted assays.

Choosing the right non-target species permitted the experimental verification and optimization of amplification conditions suitable to guarantee reliable results in the analysis of hive associated matrices. The proven exclusivity of the qPCR test optimized in this study toward these species and the sensitivity reached indicated the applicability of the method for direct analysis of honey and hive debris for surveillance and risk assessment purposes. The DNA targeted qPCR tests can also detect non-viable spores. It is not easy to ascertain this for *P. larvae* by referring to plate counts cannot serve as a reliable point of comparison to estimate this bias given since most *P. larvae* strains have a reduced germination capacity in culture media [11]. Nevertheless, the quantification of all spores present can give an estimate of AFB outbreak risk, so it is opportune to carry out further studies to establish the correlation between qPCR based quantification and AFB prevalence.

Inclusivity had to be re-assessed since most of the qPCR methods previously proposed for *P. larvae*, all targeted on the 16S rRNA gene, were developed before the acquisition of genome sequences and numerous 16S rRNA gene sequences from many *P. larvae* strains isolated all over the world. The alignment of all the *P. larvae* 16S rRNA gene sequences from the public domain database allowed to identify the primers with a perfect annealing with all strains and with potential to allow the detection of all field strains.

## 5. Conclusions

This study presents an evaluation of inclusivity and exclusivity of qPCR protocols previously proposed for the identification of *P. larvae* and the definition of a reliable test for quantification of *P. larvae* spores in honey and hive debris for AFB surveillance. The in silico and experimental evaluation resulted in the improvement of specificity for one of the existing qPCR tests and in the design of a more sensitive method derived from the latter. The qPCR protocol assessed can be adopted in standard procedures to reliably quantify *P. larvae* spores, thus estimating AFB prevalence and outbreak risk before the manifestation of clinical signs and allowing to prevent the spread of the etiological agent to other hives or apiaries from heavily infected ones. Moreover, the qPCR protocol can be used in alternative to the time-consuming cultural methods that usually give an underestimation of *P. larvae* spore load.

This investigation indicated that the choice of a diagnostic qPCR assay should be done after ascertaining that appropriate non-target species were used to ensure the specificity and after verifying inclusivity of the already described primer pairs on the basis of numerous newly acquired sequence data from organisms belonging to the target species.

## Figures and Tables

**Table 1:** Oligonucleotides designed in this study and respective positions in the 16S rRNA gene of the *P. larvae* type strain ATCC 9545, GenBank acc. CP019687, locus tag BXP28_01730.

Label	Sequence 5′→3′	Nucleotide Positions
PLAup	TTCGGGAGACGCCAGGTTA	323279–323297
PLAup2	KKTYYYTTCGGGAGACGCCA *	323273–323292
PLAdw	CTTTCATGACTTCTTCATGCGAAG	323387–323410

* According to the IUPAC code, the ambiguous primer positions have the following meaning: Y (C, T), K (T, G).
